# How visual illusions illuminate complementary brain processes: illusory depth from brightness and apparent motion of illusory contours

**DOI:** 10.3389/fnhum.2014.00854

**Published:** 2014-10-28

**Authors:** Stephen Grossberg

**Affiliations:** ^1^Center for Adaptive Systems, Graduate Program in Cognitive and Neural Systems, Center for Computational Neuroscience and Neural TechnologyBoston, MA, USA; ^2^Department of Mathematics, Boston UniversityBoston, MA, USA

**Keywords:** visual illusion, brightness perception, depth perception, motion perception, formotion perception, apparent motion, filling-in, illusory contours

## Abstract

Neural models of perception clarify how visual illusions arise from adaptive neural processes. Illusions also provide important insights into how adaptive neural processes work. This article focuses on two illusions that illustrate a fundamental property of global brain organization; namely, that advanced brains are organized into parallel cortical processing streams with computationally *complementary* properties. That is, in order to process certain combinations of properties, each cortical stream cannot process complementary properties. Interactions between these streams, across multiple processing stages, overcome their complementary deficiencies to compute effective representations of the world, and to thereby achieve the property of *complementary consistency*. The two illusions concern how illusory depth can vary with brightness, and how apparent motion of illusory contours can occur. Illusory depth from brightness arises from the complementary properties of boundary and surface processes, notably boundary completion and surface-filling in, within the parvocellular form processing cortical stream. This illusion depends upon how surface contour signals from the V2 thin stripes to the V2 interstripes ensure complementary consistency of a unified boundary/surface percept. Apparent motion of illusory contours arises from the complementary properties of form and motion processes across the parvocellular and magnocellular cortical processing streams. This illusion depends upon how illusory contours help to complete boundary representations for object recognition, how apparent motion signals can help to form continuous trajectories for target tracking and prediction, and how formotion interactions from V2-to-MT enable completed object representations to be continuously tracked even when they move behind intermittently occluding objects through time.

“Reality is merely an illusion, albeit a very persistent one.”

Albert Einstein

## Introduction

### Illusions arise from adaptive processes of a complementary brain

Neural models of perception have begun to explain how visual illusions arise from neural processes that play an adaptive role in achieving the remarkable perceptual capabilities of human and primate visual systems (e.g., Grossberg, 1994, 1997, 2008, 2014; Pinna and Grossberg, 2005, 2006; Tanca et al., 2010; Grossberg and Pinna, 2012; Cao and Grossberg, 2014). Indeed, these models show that there is a precise mechanistic sense in which all visual percepts are, at least in part, visual illusions. They do this by showing how illusions can arise from brain processes that reorganize and complete perceptual representations from the noisy data received by our retinas. These processes include boundary and surface representations that are completed over the retinal blind spot and veins, leading to conscious percepts of continuous forms, even at positions where the input signals are occluded by the blind spot or retinal veins. Many completed representations may look “real,” whereas others, whose combinations of boundary and surface properties are unfamiliar, may look like illusions.

Percepts that observers identify as illusions may arise from different brain processes, including: completion of perceptual groupings and filling-in of surface lightnesses and colors, leading to percepts of 3D form; transformation of ambiguous motion signals into coherent percepts of object motion direction and speed; and interactions between the form and motion cortical processing streams to generate percepts of moving-form-in-depth.

This article focuses on two illusions that illustrate a fundamental property of global brain organization; namely, that advanced brains are organized into parallel cortical processing streams with computationally *complementary* properties (Grossberg, 2000). In order to process certain combinations of properties, each cortical stream cannot process complementary properties. Interactions between these streams, across multiple processing stages, overcome their complementary deficiencies to compute effective representations of the world. Said in another way, these interactions convert computations that obey complementary laws into a consistent percept, hereby achieving the property of *complementary consistency*.

## Methods and results

### Brighter kanizsa squares look closer

**Figure 1:**
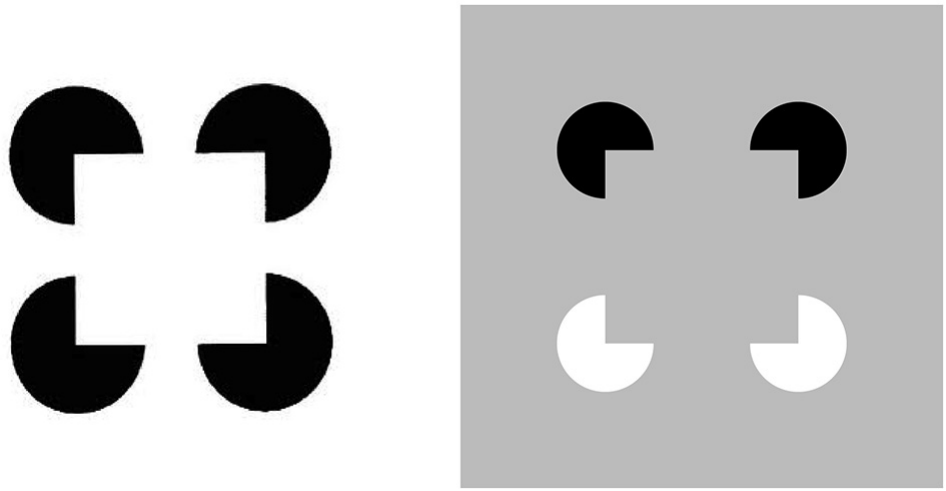
**Kanizsa square (left panel) and reverse-contrast Kanizsa square (right panel)**. The Kanizsa square appears brighter than its background due to the brightness induction by the four black pac man figures. In contrast, the reverse-contrast Kanizsa square may be recognized, but not seen, if the brightness induction by the black-to-gray pac man inducers balances the darkness induction due to the white-to-gray pac man inducers after filling-in.

**Figure 2:**
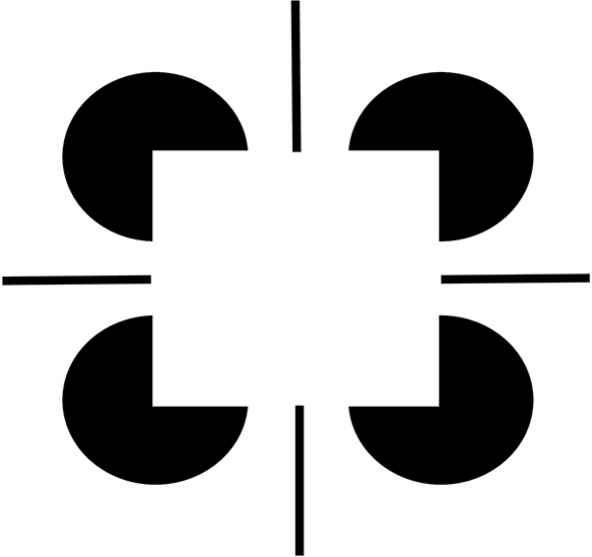
**Brighter Kanizsa squares look closer**. A Kanizsa square can look brighter when more brightness inducers exist, such as the four black lines in addition to the wide pac man inducers. As the filled-in brightness of the square increases due to more inducers, the square appears to be more separated in depth from its inducers. In addition, the pac men are recognized as partially occluded disks that are amodally completed behind the square.

Both of the visual illusions that are discussed herein use Kanizsa squares to illustrate how computationally complementary processes interact to achieve complementary consistency (Figure 1). In one such illusion, Kanizsa squares are made to look brighter by adding more inducers of the emergent illusory square. This can be done, for example, by increasing the length of the pac man inducers, or by adding some extra lines perpendicular to the illusory square between pairs of pac men (Figure 2). Remarkably, as the Kanizsa square looks brighter, it also looks closer (Kanizsa, 1955, 1974; Bradley and Dumais, 1984; Purghé and Coren, 1992). That is, a brighter square appears to be closer to the observer than its inducers, which are perceived to be partially occluded circular disks and horizontal and vertical lines that lie partially behind the square. Why do brighter Kanizsa squares look closer? In order to understand how this happens, we first need to review how boundaries and surfaces, whether “real” or “illusory,” are generated by the visual cortex.

#### Complementary properties of boundary completion and surface filling-in

**Figure 3:**
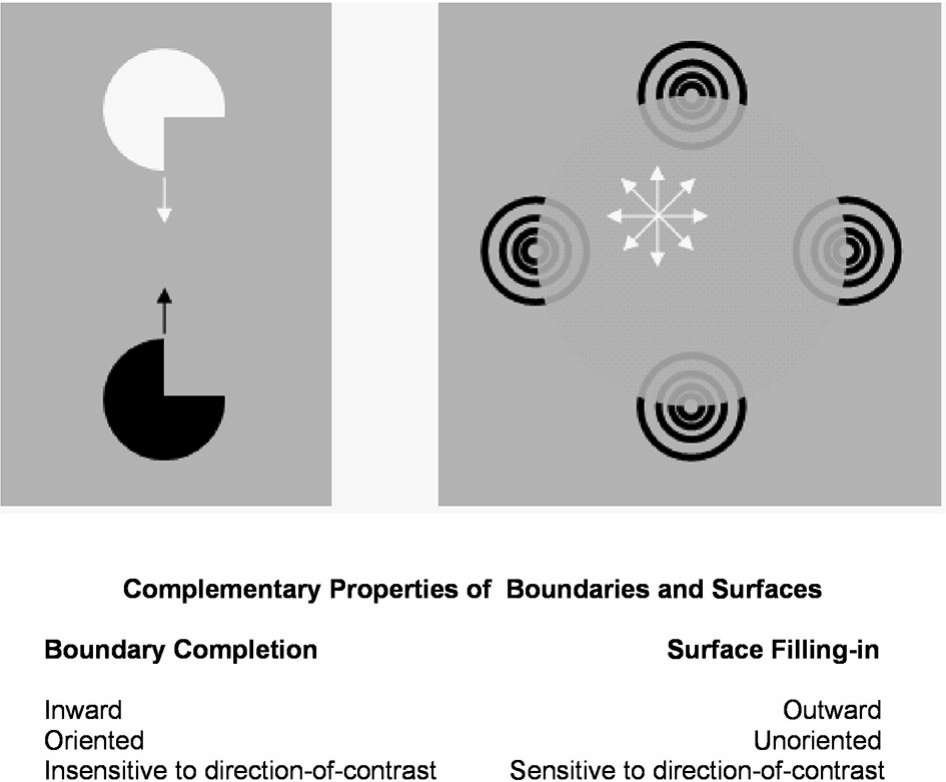
**Boundary completion and surface filling-in obey computationally complementary laws**. Boundaries complete *inwardly* in an *oriented* manner in response to pairs or greater numbers of inducers. Boundary completion also pools across opposite contrast polarities, and thus occurs in a manner that is *insensitive* to contrast polarity. As a result, “all boundaries are invisible.” In contrast, surface filling-in spreads *outwardly* from each feature contour inducer in an *unoriented* manner and does not pool opposite contrast polarities, hence is *sensitive* to contrast polarity. As a result, all conscious percepts of visual qualia are surface percepts, including percepts of such seemingly simple stimuli as dots or lines, which also generate boundary groupings that contain filling-in of their surface brightnesses and/or colors; cf., simulations in Grossberg and Mingolla (1985b).

Figure 3 summarizes the complementary properties whereby boundary groupings are completed and surfaces are filled-in with brightness or color. These properties are more thoroughly described in a series of earlier articles; e.g., Grossberg (1994, 1997, 2003). They are briefly reviewed here for completeness.

**Figure 4:**
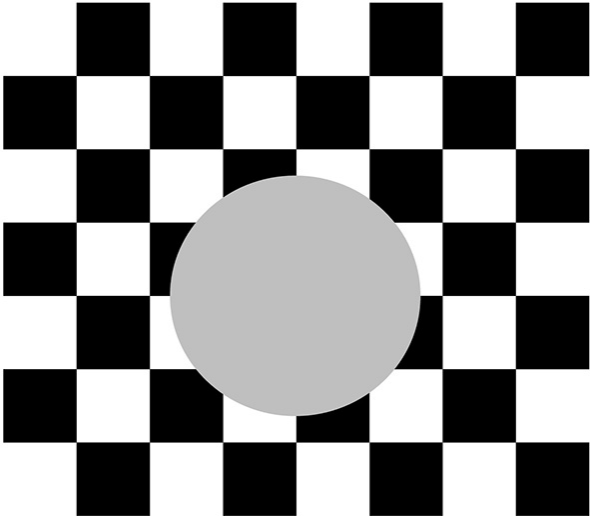
**Example of boundary polarity pooling**. The relative contrasts along the perimeter of the circular disk reverse polarity periodically. Because boundaries pool opposite contrasts at each position, they can form along the entire perimeter of the disk, and more generally around objects in front of textured backgrounds whose relative polarities shift along the object bounding contours.

All perceptual boundaries, like the Kanizsa square, are completed *inwardly* between pairs or greater numbers of inducers. This completion process proceeds in an *oriented* fashion, just as pairs of collinear pacman edges in Figure 1 induce completion of a colinear illusory contour between them. Boundaries are also *insensitive* to contrast polarity, in the sense that they pool input signals over opposite contrast polarities at each position. This last property is illustrated by a reverse-contrast Kanizsa square (Figure 1). Polarity-pooling along a boundary enables the boundary to form along the entire bounding contour of a surface that lies in front of a background whose relative contrasts reverse along the boundary's perimeter (Figure 4). The pooling property led to the prediction that “all boundaries are invisible” (Grossberg, 1984, 1994) since, by pooling over opposite contrast polarities at each position, boundaries lose the ability to represent a visible contrast difference.

**Figure 5:**
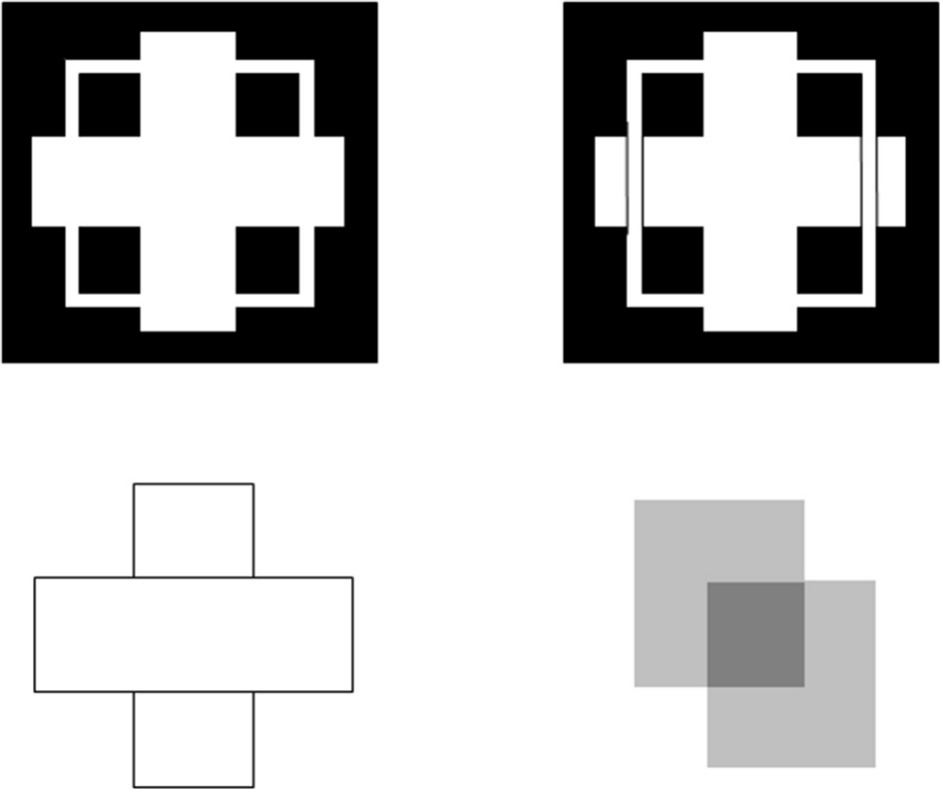
**Examples of figure-ground perception**. The two figures in the top row illustrate Kanizsa stratification. In the left panel, the white cross appears in front of the square border most of the time. The white in positions where the cross occludes the square appears to belong to the cross, and is in front of the square, which is amodally completed behind it. On occasion, the percept flips with the square appearing in front of the cross. Then the white area that previously belonged to the cross appears to belong to the square, with the cross amodally completed behind it. In the right panel, even when the extra black vertical lines force the vertical square bar to always appear in front of the cross, the horizontal branches of the square are amodally recognized behind the vertical bars of the cross, leading to a percept of a square that is bent in depth. This latter result is incompatible with a Bayesian statistics account of what the percept should look like based upon the high probability of experiencing flat squares in the world. These percepts are explained in Grossberg (1997) and simulated in Kelly and Grossberg (2000). In the bottom row (left panel), the two small rectangles are recognized as an amodally completed vertical rectangle behind the horizontal bar. This illustrates amodal completion of recognition without seeing, as do the two stratification figures. This percept, and its variants when the relative contrasts of the rectangles and background are varied, is explained in Grossberg (1997). The remaining figure in the lower right panel illustrates bistable transparency, whereby the percept of an upper left square as a transparent film in front of a lower right square alternates with the percept of a lower right square as a transparent film in front of an upper left square. This percept, as well as unimodal transparency and no transparency cases, is explained and simulated in Grossberg and Yazdanbakhsh (2005).

There are many reasons why boundaries need to be completed. One important reason is to complete boundaries at positions that do not receive retinal inputs because they occur in the region of the retinal blind spot. Another reason is to complete boundaries of partially occluded objects behind their occluders (Figure 5). Both types of completion in the visual cortex facilitate recognition of the completed boundaries at the higher processing levels of the inferotemporal cortex and beyond.

**Figure 6:**
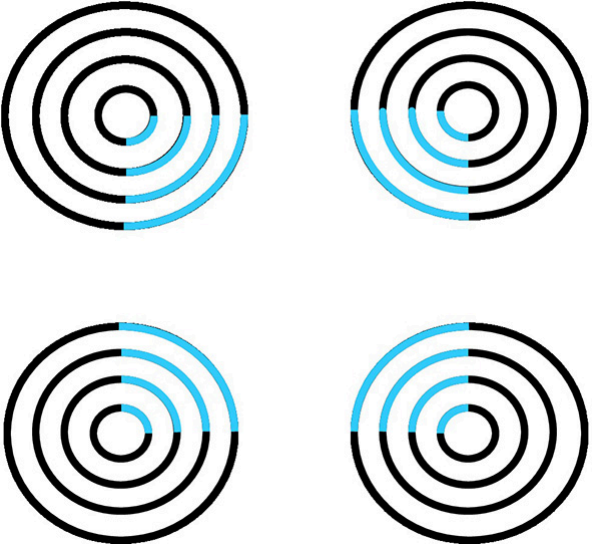
**Neon color spreading**. The blue color in the blue arcs spreads throughout the illusory square. This percept is explained and simulated in Grossberg and Mingolla (1985a).

In contrast, surface filling-in proceeds *outwardly* from its inducers in an *unoriented* fashion until it hits a boundary or dissipates due to its spatial spread, as in the percept of neon color spreading in Figure 6. Filling-in occurs in networks that are called Filling-In-DOmains, or FIDOs. There are multiple FIDOs to enable filling-in of multiple opponent colors (red-green, blue-yellow) and achromatic brightnesses (light-dark) at multiple depths. In each of these FIDOs, filling-in spreads out from feature contours that that are computed during a process of “discounting the illuminant.” Feature contours are computed at positions where luminance or color contrasts change quickly enough across space. Such positions often occur along a surface's boundary contours, which are also sensitive to contrast changes, but use different computations. Feature contours compute brightness and color signals that are significantly freed from contamination by varying illumination levels. They can do this because the contrast changes where they are computed are due primarily to changes in the reflectances of the underlying objects, whereas the illumination level changes little, if at all, across such a contrast change. Filling-in spreads the illuminant-discounted feature contour signals across the surface until they hit the boundary contours that enclose the surface. The percept of neon color spreading in Figure 6 illustrates how the square illusory contour boundary can prevent the spreading blue color from crossing it. Unlike boundary completion, filling-in is *sensitive* to contrast polarity, consistent with the prediction that “all visible qualia are surface percepts.”

These properties of boundaries and surfaces are manifestly complementary: inward vs. outward, oriented vs. unoriented, insensitive vs. sensitive (Figure 3).

Complementarity of boundary and surface processing is important for the success of each process. For example, filling-in needs to be unoriented so that it can cover an entire surface. On the other hand, the unoriented flow of brightness can only be efficiently contained by an oriented boundary. Likewise, a seeing process cannot efficiently build boundaries around objects in front of textured backgrounds. Both types of process are needed for either process to work well. Moreover, both types of process need to interact to overcome each other's complementary deficiencies.

#### Anatomical substrates of boundaries and surfaces in visual cortex

**Figure 7:**
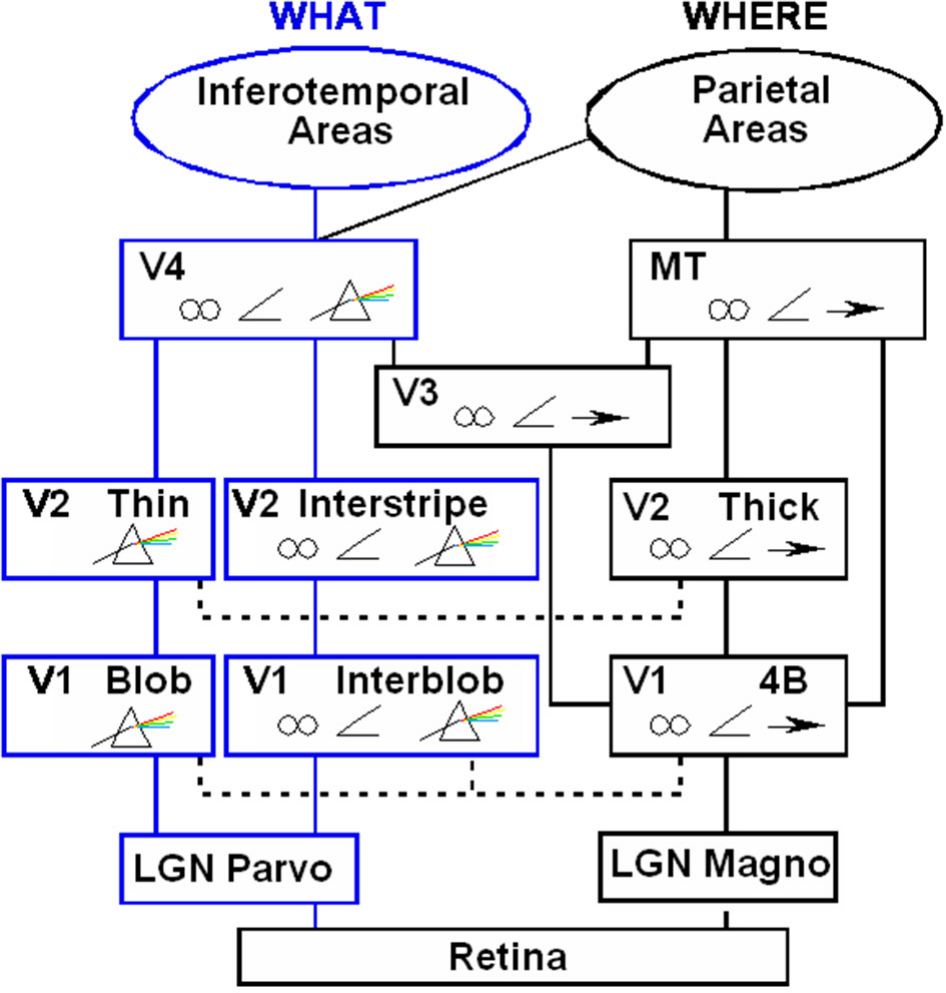
**Schematic diagram of anatomical connections and neuronal selectivities of early visual areas in the macaque monkey**. LGN, Lateral Geniculate Nucleus; V1, striate visual cortex; V2, V3, V4, MT, prestriate cortical areas. The boundary stream goes through the blobs and thin stripes to cortical area V4 and inferotemporal areas. The surface stream goes through interblobs and interstripes to V4. The motion stream goes through V1 and MT to the parietal areas. Prism = wavelength selectivity, angle symbol = orientation selectivity, spectacles = binocular selectivity, and right-pointing arrow = selectivity to motion in a prescribed direction. [Reprinted with permission from DeYoe and Van Essen (1988)].

Where are these complementary processes represented in the brain? Much evidence suggests that they are carried out by parallel processing stream in the visual cortex. Figure 7 illustrates how visual signals activate the light-sensitive retinas within our eyes. The retinas, in turn, send signals to the lateral geniculate nucleus, or LGN. Output signals from the LGN branch out and activate several parallel subsystems of the visual cortex.

Two of these streams proceed from the parvocellular LGN to regions of the first cortical stage, called area V1 in monkeys and area 17 in cats. One of these streams goes through the *blobs* of V1. The blobs are highly active metabolically and therefore light up when probed by a chemical marker called cytochrome oxydase. The blobs project, in turn, to the *thin stripes* of the prestriate cortex, in area V2 in monkeys and area 18 in cats. The thin stripes then project to prestriate area V4. A neural theory, called FACADE (Form-And-Color-And-DEpth) theory Grossberg (1987, 1994, 1997) predicts that the LGN→blob→thin stripe→V4 processing stream generates visual *surface* representations, and that the parallel LGN→interblob→interstripe→V4 processing stream generates visual *boundary* representations. Let us call these streams the blob and interblob streams, respectively. Subsequent experiments have supported this prediction (e.g., Elder and Zucker, 1998; Rogers-Ramachandran and Ramachandran, 1998; Lamme et al., 1999), and many vision scientists now routinely use the boundary/surface distinction to interpret their experiments.

#### Boundary/surface vs. orientation/color in cortical streams

Other investigators, notably Livingstone and Hubel (1984), made related, but conceptually distinct, proposals. They suggested that the blob stream computes “color” and the interblob stream computes “orientation,” rather than surfaces and boundaries. These two proposals lead to different predictions. In particular, a boundary system can complete boundaries—both “real” and “illusory”—over positions in space that receive no inputs, let alone oriented inputs; and a surface system can generate filled-in representations of figure-ground relationships that do not directly represent the local brightnesses and colors of a scene. In particular, FACADE theory predicts why completed boundaries and filled-in surfaces of the occluded parts of objects are often amodal, or invisible, including the Kanizsa stratification examples in Figure 5, even though they can have profound effects on object recognition. Grossberg (1994, 1997) and Kelly and Grossberg (2000) have used FACADE theory to explain and simulate a number of challenging figure-ground percepts that include amodally completed, partially occluded objects, including examples of stratification.

Occluders do not always render occluded object parts invisible. Percepts of transparency illustrate how this can happen. Figure 5 includes an example of the particularly interesting, and challenging, percept of bistable transparency. Here, in response to a fixed 2D image, the brain perceives either of two alternating percepts of a transparent surface “behind” its occluding surface. The surfaces that are perceived to be occluding or occluded alternate through time. Grossberg and Yazdanbakhsh (2005) have provided mechanistic explanations of when transparency does or does not occur, including simulations of bistable transparency, in terms of the 3D LAMINART model (Grossberg, 1999; Cao and Grossberg, 2005). The 3D LAMINART model extends FACADE theory to propose how identified cells in the laminar circuits of visual cortex interact to generate visual percepts.

#### Complementary consistency: surface contours and boundary-mediated surface capture

**Figure 8:**
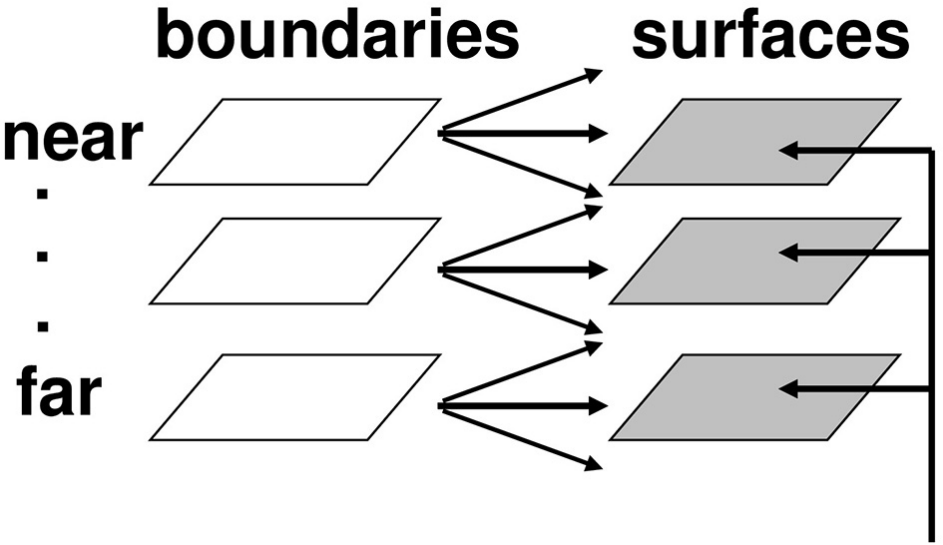
**Multiple depth-selective boundary representations regulate filling-in of surface representations within multiple depth-selective Filling-In DOmains**. Brightness or color feature contour inputs are topographically distributed across multiple depths (vertical arrows) before being captured by boundaries (horizontal and oblique arrows) that are positionally aligned with them. See Grossberg (1994) for a more complete description of this surface capture process.

**Figure 9:**
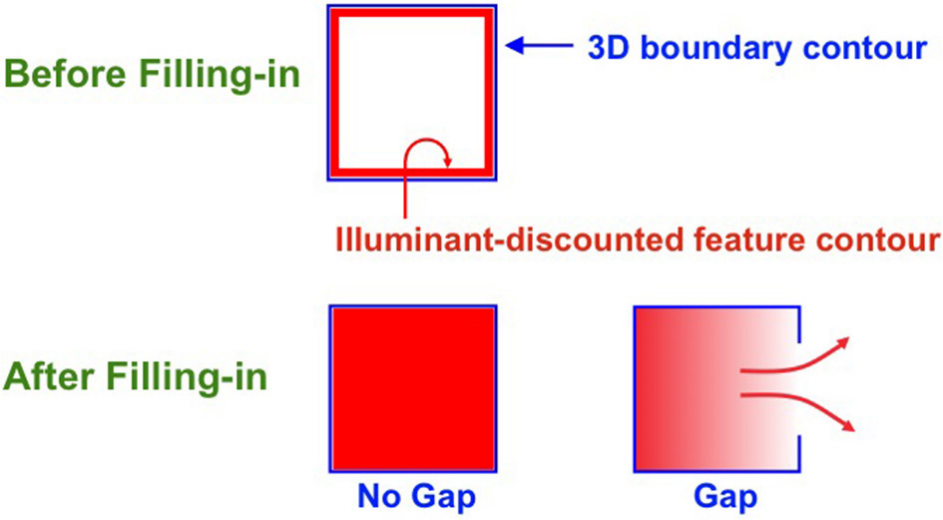
**Filling-in closed and open boundaries**. The top row illustrates how, at a prescribed depth, a closed boundary contour abuts an illuminant-discounted feature contour. When this happens, the feature contour can fill-in within the closed boundary. The bottom row (left panel) depicts how filling-in of the feature contour is contained by this closed boundary contour, thereby generating large contrasts in filled-in activity at positions along the boundary contour. Contrast-sensitive surface contour output signals can then be generated in response to these large contrasts. The bottom row (right panel) depicts a boundary contour that has a big hole in it at a different depth. Feature contours can spread through such a hole until the filled-in activities on both sides of the boundary equalize, thereby preventing contrast-sensitive surface contour output signals from forming at such boundary positions.

In order to represent a 3D scene, there are multiple boundary and surface representations, each sensitive to a different range of depths from an observer. FACADE theory predicts how 3D boundary signals are topographically projected from where they are formed in the V2 interstripes to the surface representations in the V2 thin stripes (Figures 7, 8). These boundaries act both as *filling-in generators* that initiate the filling-in of surface lightness and color at positions where the corresponding boundary contour and feature contour signals are aligned, and as *filling-in barriers* that prevent the filling-in of lightness and color from crossing object boundaries (Grossberg, 1994). If a boundary at a given depth is closed, it can contain the filling-in of an object's lightness and color within it (Figure 9). If, however, the boundary at a different depth has a sufficiently big gap in it, then surface lightness and color can spread through the gap and surround the boundary on both sides, thereby equalizing the contrasts on both sides of the boundary. Only a closed boundary can contribute to the final visible 3D percept.

**Figure 10:**
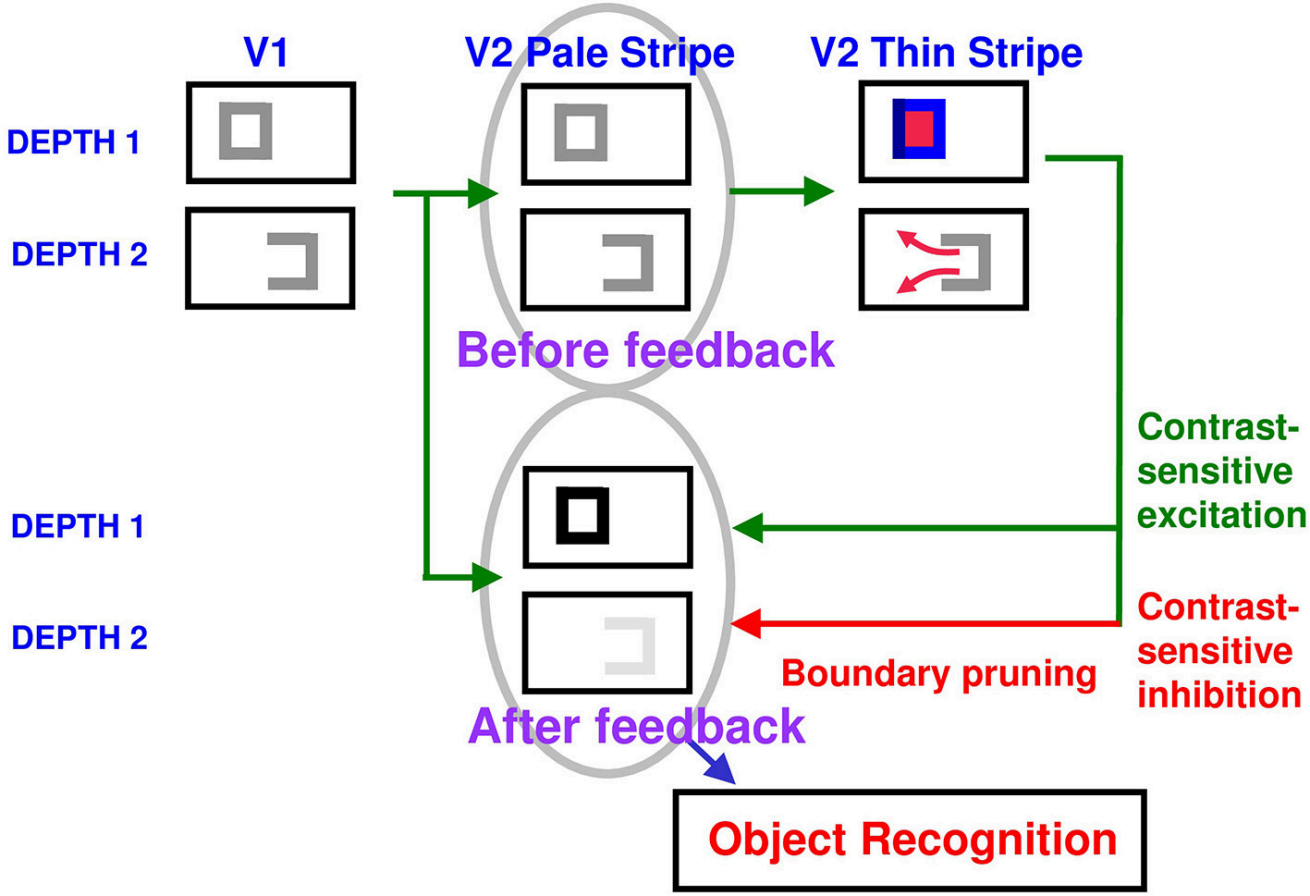
**Surface contour formation**. A closed boundary can form at Depth 1 by combining a binocular vertical boundary at the left side of the square with three monocular boundaries that are projected along the line of sight to all depths. Surface contour output signals can thus be generated by the FIDO at Depth 1, but not the FIDO at Depth 2. The Depth 1 surface contours excite, and thereby strengthen, the boundaries at Depth 1 that controlled filling-in at Depth 1. These surface contours also inhibit the redundant boundaries at Depth 2 at the same positions. As a result, the pruned boundaries across all depths, after the surface contour feedback acts, can project to object recognition networks in inferotemporal cortex to facilitate amodal recognition, without being contaminated by spurious boundaries. See Fang and Grossberg (2009) for simulations of how this process works in response to random dot stereograms.

How do closed boundaries help to form a visible 3D percept? In addition to the boundary-to-surface interactions that act as filling-in generators and barriers, there are also surface-to-boundary feedback interactions from filled-in surfaces in V2 thin stripes to the boundaries in V2 interstripes (Figure 10). This feedback takes the form of *surface contour* signals that are generated by contrast-sensitive on-center off-surround networks whose inputs are the filled-in surface activities within each FIDO. The inhibitory connections of each network's off-surround act across position and within depth in order to generate contrast-sensitive output signals from each FIDO. Surface contour signals are therefore generated at the positions where sufficiently large changes in brightness or color occur within successfully filled-in surface regions. Consequently, if the object surface in a FIDO is surrounded by a closed boundary, then there is typically a discontinuity in the contrasts across the object boundary. These positions typically occur at salient features on an object's surface. Surface contour signals are not generated at boundary positions near a big boundary gap, since lightnesses and colors can then be equal, hence have zero contrast, on both sides of the boundary due to filling-in.

The surface contour output signals generate feedback signals to the boundary representations that induced them. These feedback signals are also delivered to the boundary representations via an on-center off-surround network. However, the inhibitory connections of surface contour networks act within position and across depth (Figure 10). The on-center signals strengthen the boundaries that generated the successfully filled-in surfaces, whereas the off-surround signals inhibit spurious boundaries at the same positions but farther depths. This inhibitory process is called *boundary pruning*. Surface contour signals hereby achieve complementary consistency by strengthening consistent boundaries and pruning inconsistent boundaries.

A crucial property of boundary pruning is that it eliminates boundaries at depths that do not support visible filled-in surfaces. Boundary pruning is thus part of the process of *surface capture* whereby feature contours can selectively fill-in visible surface qualia at depths where binocular fusion of object boundaries can successfully occur, and can thereby create closed boundaries that can contain the filling-in process. Surface contour and boundary pruning signals hereby work together to generate 3D percepts based on successfully filled-in surface regions.

Remarkably, by eliminating spurious boundaries, the off-surround signals also initiate figure-ground separation. They do so by enabling occluding and partially occluded surfaces to be separated onto different depth planes, after which partially occluded boundaries and surfaces can be amodally completed behind their occluders. See Fang and Grossberg (2009), Grossberg (1994), and Kelly and Grossberg (2000) for further details and simulated figure-ground percepts. See Bakin et al. (2000) for experiments in monkeys that describe how amodal contour completion and surface capture may occur in V2.

#### How brighter-thus-closer kanizsa squares illustrate complementary consistency

With this background of FACADE theory concepts, we can now explain, as a manifestation of neural mechanisms that are needed to realize adaptive visual properties, the curious fact that brighter Kanizsa squares look closer.

We first must note that a Kanizsa square's perceived brightness is an emergent property that is determined after *all* brightness and darkness inducers fill-in within the square; e.g., Figures 1, 2. The emergent brightness of the square as a whole can only then influence the square's perceived depth. In particular, the computation that leads the square surface to appear closer can only occur after filling-in occurs within the surface FIDO representations.

Within FACADE theory, the perceived depth of a surface is controlled by the boundaries that act as its filling-in generators and barriers (Figure 8), since these boundaries select the depth-selective FIDOs within which filling-in can occur, and thereby achieve surface capture. These boundaries, in turn, are themselves finally selected after surface-to-boundary surface contour feedback eliminates redundant boundaries that cannot support successful filling-in (Figure 10). But surface contour feedback signals have precisely the properties that are needed to explain why brighter Kanizsa squares look closer!

**Figure 11:**
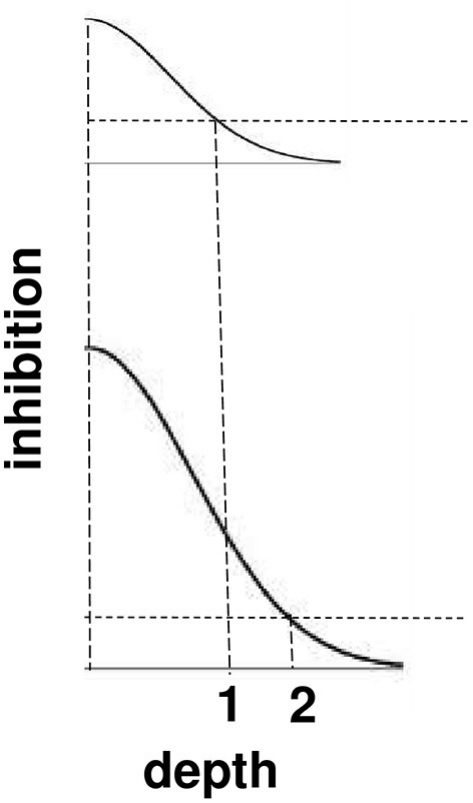
**Surface contour inhibition of boundaries across depth**. A cross-section of the inhibitory off-surround across depth that is caused by surface contour outputs. The top curve shows the inhibitory signals in response to a less bright Kanizsa square. The bottom curve shows the inhibitory signals in response to a more bright Kanizsa square. The numerals 1 and 2 indicate one of the depths where the inhibitory signals are equal. This illustrates how the brighter Kanizsa square can inhibit boundaries at more depths between that of the Kanizsa square and its inducers, thereby making the brighter square stand out more in depth.

Why this is true can be seen from Figure 11. Recall that the off-surround of surface contour signals occurs within each position and across depth. As in essentially all off-surround networks, the strength of inhibition decreases with the distance from the source cell. In the present case, “distance” translates into a depth difference. Thus, the strength of the inhibitory signals *decreases* as the depth difference *increases* between the depth of the surface that generates the surface contour signals and the recipient boundaries.

The brightness of a Kanizsa square increases with the amplitude of the filled-in activity within the square. A larger activity creates larger inhibitory signals at each position. These signals are multiplied by the strengths of the inhibitory connections from the signal source to the recipient boundary at the same position but a different depth. Due to the decrease in size of the inhibitory connections across depth, these net signals also get smaller as the depth difference increases. The top curve in Figure 11 represents the total strength of these inhibitory signals across depth at a lower level of brightness, and the bottom curve represents the total inhibitory signals across depth at a higher level of brightness. The numbers 1 and 2 illustrate that the same level of inhibition is achieved at a larger depth difference in response to a brighter Kanizsa square. In other words, a larger number of boundary depths are inhibited by a brighter square than a dimmer one, so that the depths of the boundaries that survive well enough to represent the background are further away in depth than those that survive in response to a dimmer square. In short, brighter Kanizsa squares look closer, relative to their backgrounds, than dimmer ones.

### Apparent motion of illusory contours

#### Complementary processing streams for form and motion perception

**Figure 12:**
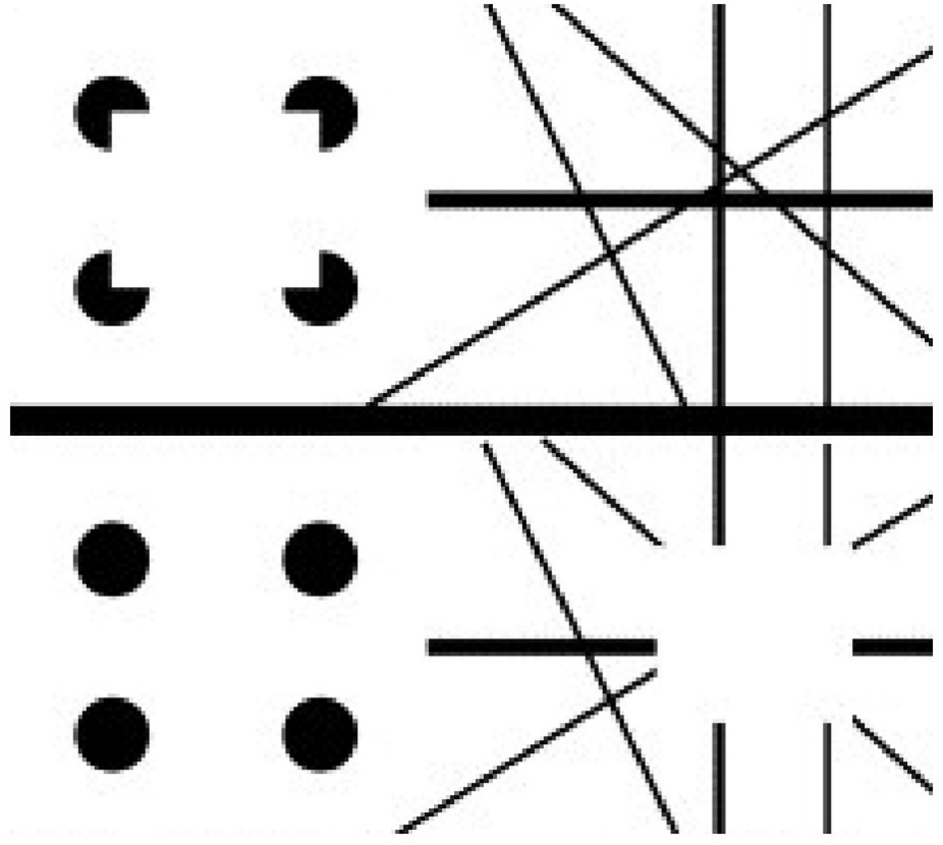
**Apparent motion of illusory contours**. These images demonstrate that apparent motion of illusory contours arises through the interaction of the static illusory contours via formotion interactions that are predicted in Grossberg (1991) to occur from cortical area V2 to cortical area MT. Frame 1 (row 1) is temporally followed by Frame 2 (row 2) and conversely. [Adapted with permission from Ramachandran (1985)].

The second illusion to be explained is the apparent motion of illusory contours (Figure 12) that was reported by Ramachandran et al. (1973) and Ramachandran (1985). This is a “double illusion” in the sense that it combines the formation of illusory contours in the form processing stream and long-range apparent motion in the motion processing stream. In particular, the 3D FORMOTION model (Grossberg, 1991, 1998; Francis and Grossberg, 1996; Baloch and Grossberg, 1997; Chey et al., 1997; Grossberg et al., 2001; Berzhanskaya et al., 2007) explains this illusion in terms of how the form processing stream from V1-to-V2 generates illusory contours *and* interacts with the motion processing stream from V1-to-MT via V2-to-MT interactions. This *formotion* interaction is predicted to overcome the computationally complementary properties of the form and motion streams acting alone, properties that will be summarized below. Moving-form-in-depth can hereby be computed for purposes of object tracking via MT-to-MST-to-PPC interactions. Apparent motion of illusory contours illustrates this property, albeit in response to such a reduced visual stimulus that the percept seems more like a curiosity than an illustration of a fundamental competence that is needed for survival.

To generate this percept, two visual frames alternate through time. In one frame, there are four pac man figures that induce a Kanizsa square percept. To the right of the pac men is a random array of intersecting oriented lines. In the second frame, the pac men are replaced by four filled circles and the lines are deleted in the region of an imaged square, again producing an illusory square percept. By this construction, there are no square-inducing features that can be matched across the left and right images. Despite this fact, when the frames alternate through time, a percept of a square moving from left to right and back again is clearly perceived.

Mechanisms of boundary completion and surface filling-in within the form stream, such as those such as those illustrated by Figures 1–6, can be used to explain the illusory square percepts. Mechanisms of long-range apparent motion in the motion stream, responding to V2-to-MT signals from the successive images of the illusory contours in V2, can be used to explain the apparent motion of illusory contours. Many other visual illusions have also been explained using these formotion mechanisms, including Korte's laws, the line motion illusion, motion induction, and transformational apparent motion (e.g., Francis and Grossberg, 1996; Baloch and Grossberg, 1997).

#### Why do parallel form and motion cortical processing streams exist?

When an object moves across a scene, its boundary and surface representations are recreated time and time again, leading to a succession of individual object views. An immediate question arises: Is object motion just a temporal succession of static form representations? Much experimental evidence shows that object motion is *not* just a temporal succession of static form representations. In fact, the brain devotes an enormous amount of tissue to processing object motion.

These anatomical facts raise a basic question: Why has evolution evolved parallel cortical streams from V1 through V2 *and* from V1 through MT for the processing of static form and moving form, respectively? This is true despite the fact that simple cells of cortical area V1 are already sensitive to form-related properties, such as an object's orientation, as well as to motion-related properties, such as an object's direction-of-motion and temporal changes in object luminance. If simple cells can already compute properties of both object form and motion, then why did the brain need to evolve two separate processing streams to compute form and motion properties? Why is this not a huge waste of processing resources? Why could not one stream do both?

Grossberg (1991) proposed that the two streams compute complementary visual properties, and that each stream, acting alone, can compute one set of properties, but not the complementary set of properties by the same cells (Table 1). From this computational perspective, the parallel processing of static form and moving form by the What and Where streams is thus no more redundant than the parallel processing of the complementary properties of object boundaries and surfaces by the interblob and blob streams. As in the case of static boundaries and surfaces, the form and motion streams also need to interact to overcome their complementary deficiencies. These interactions between the form and motion streams can be used to clarify how the brain tracks objects moving in depth. Some of these complementary properties will now be reviewed, before using them to show how apparent motion of illusory contours can be readily explained by them.

**Table 1:** **Complementary form and motion stream computations are fused using cross-stream formotion interactions**.

	**Depth**	**Direction**
Form	Fine	Coarse
Motion	Coarse	Fine
Formotion	Fine	Fine

#### Complementary computing of orientation and direction

The discussion of boundary completion (cf. Figures 1–3, 5, 6) showed that the form system is sensitive to the *orientation* of object contrasts, and uses these oriented estimates to activate and complete object boundaries, often with the help of illusory contours. Such oriented estimates are also needed to represent objects in depth: Because the two eyes look out on the world from slightly different positions in the head, they typically register object features at different positions on their respective retinas. This difference in position is called *binocular disparity*. Binocular disparity is one of the cues used to help determine how far objects are from an observer. The binocular matching process, which is called *stereopsis*, begins in area V1 of the visual cortex. Binocular matching is highly sensitive to the orientation of the matched features, to help ensure that only features that come from the same object are matched. Thus, the form system depends on its ability to make precise estimates of feature orientation during both the earliest stages of stereopsis and the later stages of boundary completion.

**Figure 13:**
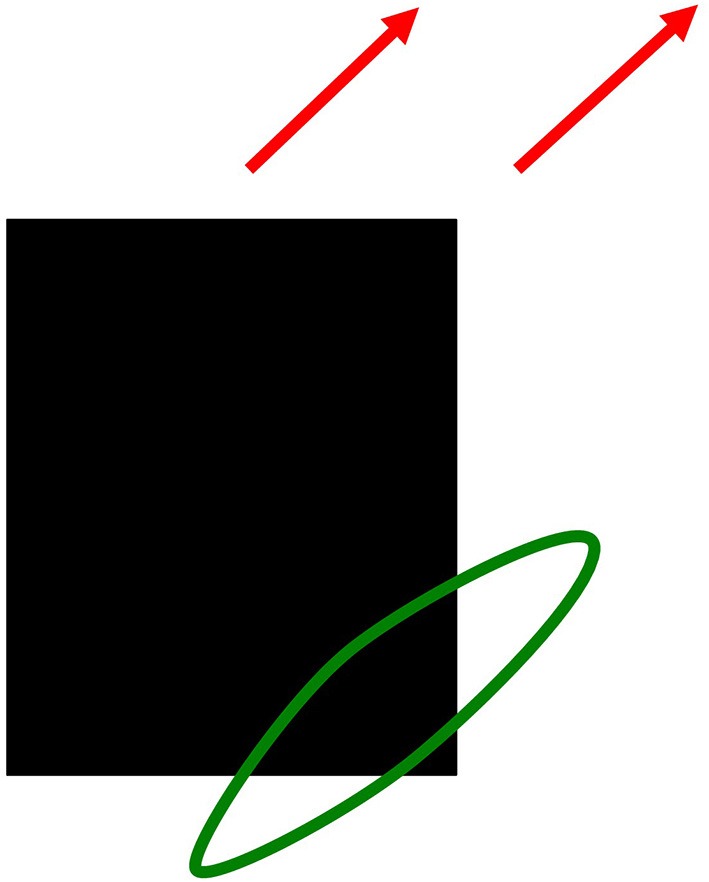
**Pooling motion signals from multiple orientations moving in the same direction with a directional long-range-filter**. A black rectangle moving diagonally up and to the right (red arrows). Motion signals from a vertical light-dark edge and a horizontal dark-light edge are pooled by a long-range directional filter to create a more precise motion direction signal.

In contrast, the motion system generates an estimate of an object's *direction* of motion. Because a single object can contain features with many different orientations that are all moving in the same direction, the motion system pools directional information that is derived from features with multiple orientations. For example, consider a rectangular black object moving diagonally upwards and to the right on a white background, as in Figure 13. At the lower right corner of the rectangle, a light-to-dark vertical edge and a dark-to-light horizontal edge both move diagonally upwards. The brain pools these two orientations, despite the fact that they have opposite contrast polarities, to estimate the direction in which the object is moving. The motion pooling process uses a filter that is big enough to accumulate directional evidence from multiple positions as an object moves in a given direction. By pooling signals from features with different orientations, but the same motion direction, the brain can compute much better estimates of an object's true direction-of-motion. Such a filter, which operates between cortical areas V1 and MT in the motion system of the Where cortical stream, is called a *long-range directional filter*.

The pooling over orientation prevents the type of orientation-specific binocular matching that is needed to generate precise estimates of object depth. Thus, the motion system, on its own, can generate precise estimates of an object's direction of motion, but at best coarse estimates of object depth, as reported by Malpeli et al. (1981), who used neurophysiological recordings under conditions where the inputs to the form system from the lateral geniculate nucleus, or LGN, were pharmacologically inhibited.

In contrast, the form system, on its own, can generate precise estimates of an object's depth, but only coarse estimates of its direction of motion. Supportive neurophysiological data, as reported by Foster et al. (1985), showed that many complex cells in V1, which have precise orientational tuning, respond to objects moving in opposite directions of motion. These kinds of considerations led to the prediction in Grossberg (1991) that the properties of the orientationally-based form system and the directionally-based motion system are computationally *complementary*.

If this is true, then it raises the following question: How does the brain use interactions between the form stream and the motion stream, which I have called *formotion* interactions, to overcome the complementary deficiencies of either stream acting alone? Grossberg (1991) predicted that one source of such formotion signals arise in area V2 of the What stream of visual cortex and are received by cells in area MT of the Where stream of visual cortex. This prediction proposed that the depth-selective boundary groupings that are computed in V2 use V2-to-MT interactions to select motion signals in MT that are consistent with them. Such selected signals could effectively represent an object's motion-direction-in-depth, and enable the brain to track the object as it moves through time.

Some experimental neuroscientists (Livingstone and Hubel, 1984, 1987; DeAngelis et al., 1998) had drawn a different conclusion from their neurophysiological data. They asserted that object depth is computed directly in MT. Their proposal raised perplexing issues about why depth is computed in MT of the motion stream, given that binocular disparity is computed by complex cells in cortical area V1 of the form stream (Figure 7) and that depthful form boundaries seem to be completed in area V2 of the form stream. The latter property is illustrated by classical data of Von der Heydt et al. (1984), who demonstrated that illusory contours form in area V2 of monkeys. My prediction that the form and motion streams compute computationally complementary properties provided a natural explanation: It proposed that the finer depth-selective properties recorded in MT are derived from V2-to-MT signals from depth-selective boundary representations in V2.

Strong support for this V2-to-MT prediction was published in Ponce et al. (2008), who did an ingenious experiment in which they cooled V2 and recorded from MT cells in monkeys. They found that, under these conditions, MT cells preserved their directional selectivity but could only code coarse depth estimates. When the cooling was reversed, MT cells coded both good directional selectivity and good depth estimates, as predicted in Grossberg (1991).

#### Long-range apparent motion and target tracking

Given that the illusory squares in response to the images in Figure 12 form in V2 and are projected to MT via V2-to-MT interactions, how do they generate a percept of apparent motion between them as the two displays in Figure 12 are alternated through time? These apparent motion signals arise from the ability to track a predator or prey animal through time when it moves at variable speed behind intermittent occluders, such as bushes and trees between the observer and the animal.

How are the intermittent views, or “flashes,” of the animal interpolated as it moves at variable speed behind and between the occluders? In particular, how are these “flashes” used to compute a continuous motion trajectory that can help to track and predict the animal's position through time? Such an interpolation process between intermittent views of a target object is a type of *long-range apparent motion*. After the form system separates the forest cover from the unoccluded “flashes” of the animal, the long-range apparent motion process can amodally complete the motion signals derived from these intermittent flashes to form a continuous motion trajectory.

I will discuss below some facts about apparent motion, and a simple mechanism that can explain them, as well as the apparent motion of illusory contours. Although these facts may at first seem functionally quite pointless, and even bizarre when studied in isolation in the laboratory, their functional importance can be understood when we consider them from the perspective of tracking a target that is moving with variable speed in the real world.

#### Apparent motion: phi and beta motion

Exner (1875) provided the first empirical evidence that the visual perception of motion was a distinct perceptual quality, rather than being merely a series of spatially displaced static percepts of a moving object. Exner did this by placing two sources of electrical sparks close together in space. When the sparks were flashed with an appropriate time interval between them, observers reported a compelling percept of a single flash moving continuously from one location to another, even though neither flash actually moved. The interstimulus interval, or ISI, is the time duration between the offset of one flash and the onset of the next flash. At very short ISIs, flashes look simultaneous and stationary. At sufficiently long ISIs, they look like successive stationary flashes, with no intervening motion percept. At some intermediate ISIs, a “figureless” or “objectless” motion called *phi motion* is perceived, wherein a sense of motion occurs without a clearly defined percept of form. A smooth and continuous motion of a perceptually well-defined form, called *beta motion*, can be seen at larger ISIs.

#### How apparent motion speed varies with flash ISI, distance, and luminance

Later discoveries about the properties of apparent motion raise philosophical as well as scientific questions. For example, a decrease in the ISI between successive flashes causes the speed of the apparent motion between them to increase just enough to interpolate the timing of the inducing flashes (Kolers, 1972). A motion percept can also smoothly interpolate flashes separated by different distances, speeding up if the ISI between the flashes is fixed as the distance between them is increased. As Kolers (1972, p. 25) noted: “large variations in distance are accommodated within a near-constant amount of time.” Motion properties also depend on the intensity of the flashes, as well as their position and timing. For example, if a more intense flash follows a less intense flash, then the perceived motion can travel backwards from the second flash to the first flash. This percept is called *delta motion* (Korte, 1915; Kolers, 1972). *Gamma motion* is the apparent expansion of the area of a flash at its onset, or its contraction at its offset (Bartley, 1941; Kolers, 1972). A similar expansion-then-contraction may be perceived when a region is suddenly darkened relative to its background, and then restored to the background luminance. *Split motion* (DeSilva, 1926) can be observed when a single flash is followed by a pair of flashes on opposite sides of the first flash. Then motion can be perceived radiating simultaneously in opposite directions from the first flash. Other curious properties include the fact that “the less you see it, the faster it moves” (Giaschi and Anstis, 1989), which means that shorter flash durations may be associated with higher judged motion speed.

#### The motion ESP problem

These discoveries raise perplexing issues concerning the brain mechanisms that generate a continuous motion percept between two stationary flashes and, thus, more generally about how the brain perceives motion in the first place: If a continuous motion seems to grow out of the first flash when the second flash occurs, then there must be some sort of long-range interaction that can link the two flashes across space. Why is this long-range interaction not perceived when only a single light is flashed? In particular, why are not outward waves of motion signals induced by a single flash?

A motion signal emerges from the location of the first flash only after the first flash terminates, indeed only after the second flash turns on some time later. How does the brain “know” that a second flash will be occurring after the first flash shuts off, so that it can create a continuous motion from the first flash to the second flash?

I like to call this the Motion ESP Problem, since it would seem that the brain needs to know in advance whether a second flash will occur, when it will occur, and in what direction it will occur, in order to create a continuous motion signal from the first flash to the second flash. Delta motion vividly illustrates this problem, since it shows how motion can be perceived from the second flash to the first flash under some conditions.

Any biologically relevant answer to the Motion ESP problem also needs to explain: How does the motion signal adapt itself to the variable distances and ISIs of the second flash? In particular, if the flashes are placed farther apart in space but the ISI remains constant, then the motion has to move faster. If the flashes remain at the same spatial separation but the ISI is decreased, then the motion again has to move faster. How can the motion signal adapt its speed to the ISI and the distance between two flashes even though such adaptation can only begin after the second flash begins?

Although these properties may appear unrealistic in the laboratory, in the real world the “flashes” may be intermittent appearances between occluding bushes and trees of a predator or prey who is moving with variable speed. The ability of apparent motion to bridge variable durations and distances enables the brain to form a continuous trajectory of an intermittently seen target as it moves with variable speed behind occluders of variable size. This trajectory can then be used by the motion system to track the predator or prey, and predict where it will be at a future time.

The above properties are a subset of those that are known about apparent motion. All the key properties have been explained and simulated by the 3D FORMOTION model. Here, only the main mechanism that helps to explain properties of long-range apparent motion will be summarized. A more complete understanding also requires an analysis of the pre-processing and post-processing mechanisms of the motion stream that includes V1, MT, MST, PPC, and PFC.

#### Long-range apparent motion arises from G-waves

Many apparent motion data can be explained using three simple mechanisms that are individually well-known to psychologists and neuroscientists (Grossberg and Rudd, 1989, 1992). When these mechanisms work together, they generate apparent motion data as emergent properties of their interaction. The mechanisms are:

1. inputs activate receptive fields that have a Gaussian shape across space;
2. responses to inputs decay gradually, indeed exponentially, through time after the inputs shut off; and
3. responses are sharpened across space by a mechanism of spatial competition, or lateral inhibition.

The second property, of temporal decay, has been shown through experiments to take quite a long time to occur, as illustrated by data published in Anstis and Ramachandran (1987). This kind of exponential decay through time is called *visual inertia*. Visual inertia can take up to a half a second to fully decay.

How do the three simple properties (1–3) work together to generate apparent motion? Remarkably, when two input flashes at different positions in space occur in succession, these mechanisms can together generate a *traveling wave* of activation that can move continuously through time between the two flashes with the same properties as the data. I have called such a wave a Gaussian-wave, or *G-wave*, because it can occur in any part of the brain that uses Gaussian receptive fields. In fact, shifts in attention through time often have the same properties as apparent motion. I have proposed that they are caused by the same type of Gaussian mechanism. Indeed, in some cases, attention shifts may be driven by the same wave of apparent motion that enables target tracking to occur. As attention shifts with the wave, it can trigger commands to the eyes and head to move accordingly.

**Figure 14:**
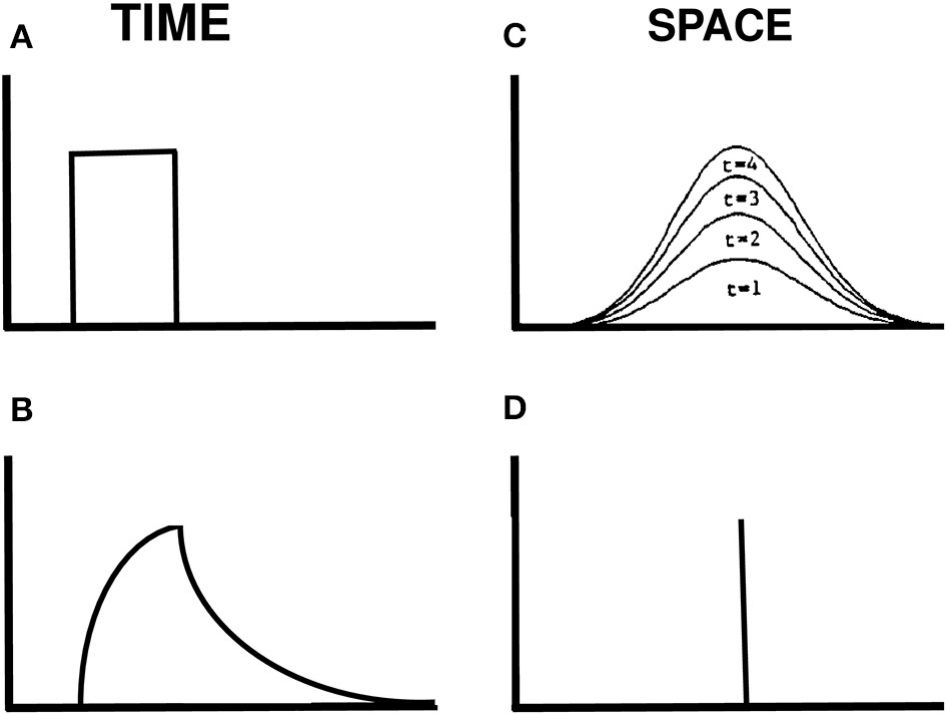
**Gaussian activation and decay in response to a single flash. (A)** Input to one position in time. **(B)** Rising and decaying activity caused by the input. **(C)** The input is filtered by a Gaussian kernel across space. Its increasing strength at four times when the input is on are shown. **(D)** The maximum of the Gaussian activity occurs at the same position at all these times.

How is a traveling wave generated by these mechanisms? Figure 14 illustrates how a spatially localized flash, which is assumed for simplicity to turn on to a fixed intensity before shutting off abruptly at a later time (Figure 14A), can cause the graded activity of the recipient cells to wax and wane through time (Figure 14B). The decaying trace looks very much like visual inertia. When this temporal profile activates the Gaussian filter across space, it generates a spatially distributed input at the next level of cells. This Gaussian profile of activation waxes and wanes through time, without spreading across space. Its waxing phase is shown in Figure 14C. This broad Gaussian activation is sharpened into a focal activation by spatial competition across these cells (Figure 14D). The position of the maximum value of this Gaussian activity profile does not move through time. If we suppose that the percept of motion covaries with this maximum value, then we can understand why a single flash does not cause a movement across space.

**Figure 15:**
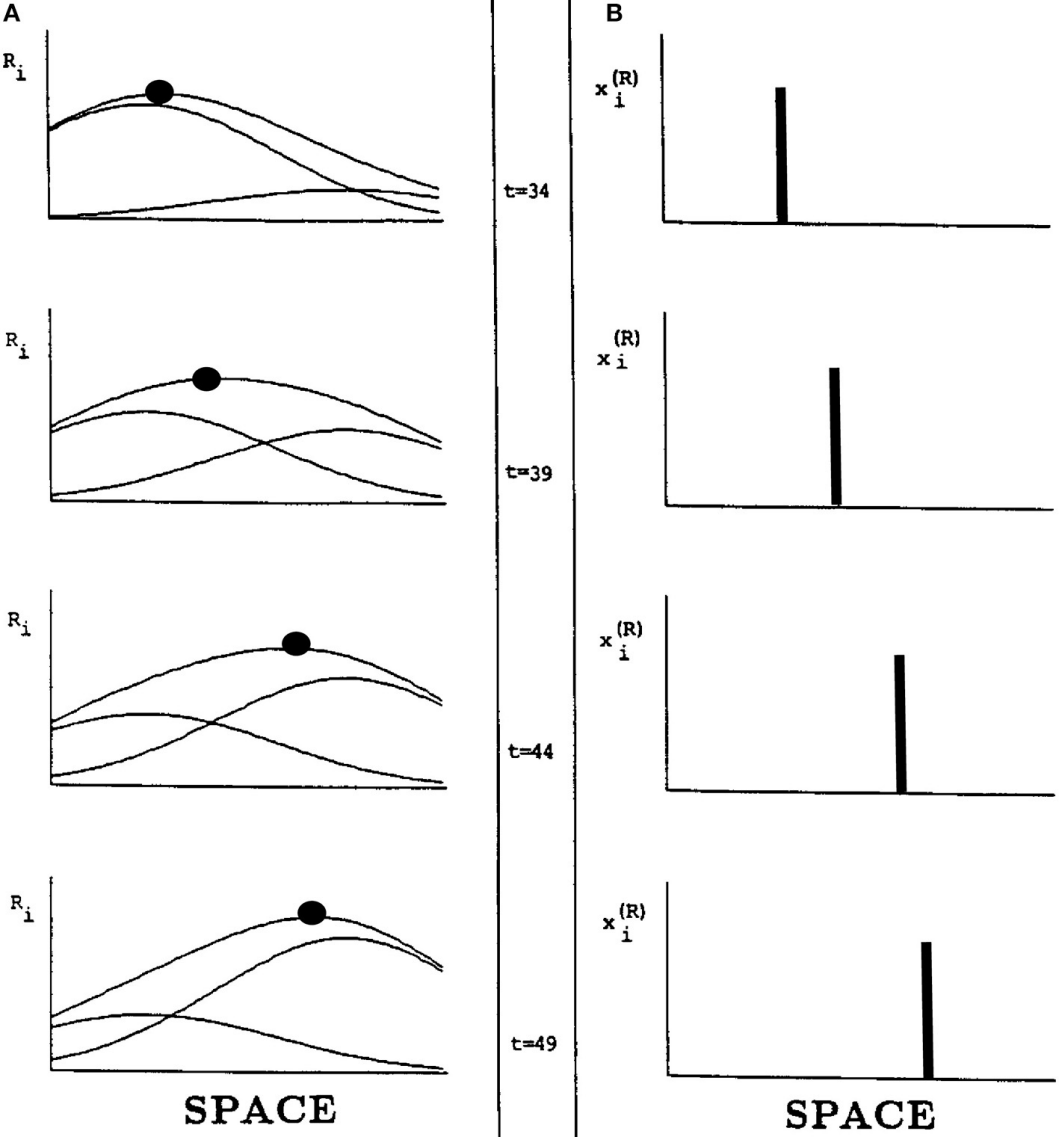
**Computer simulation of a G-wave. (A)** suppose that two successive flashes occur at times when the Gaussian activation in response to the first flash is decreasing while the Gaussian activation in response to the second flash is increasing, and the two Gaussians overlap in space. Then the maximum activity of the sum of Gaussians can create a traveling wave through time, whose maximum (black dots) moves continuously from left to right **(B)** in a way that mimmicks many properties of long-range apparent motion. [Adapted with permission from Grossberg and Rudd (1992)].

Suppose, however, that two successive flashes occur at nearby positions. Imagine that the activation in response to the flash at the first position is decaying while activation is growing in response to a flash at the second position. Under these circumstances, the *total* input from both flashes is the sum of a temporally waning Gaussian plus a temporally waxing Gaussian, as in Figure 15A. Competition selects the position of the sum's maximum activity at each time. Under appropriate spatial and temporal conditions, the maximum travels continuously through time from the position of the first flash to the position of the second flash, as in Figure 15B.

In summary, the space- and time-averaged cell responses to individual flashes do not change their positions of maximal activation through time. In this sense, nothing moves in response to a single flash. When a series of properly timed and spaced flashes is presented, however, the sum of their responses can produce a continuously moving peak of activity between the positions of the flashes. This is an emergent property of network interactions across multiple cells through time, rather than a property of any cell acting alone. The Motion ESP problem may thus be solved using the fact that the Gaussian response to the first flash is still waning—without causing a percept of motion—when the second flash occurs. The residual effect of the first flash as it combines with the waxing effect of the second flash enables the traveling wave to continuously interpolate variable ISIs and distances between the two flashes.

## Conclusion

### Illusions that reflect figure-ground separation and target tracking

In summary, illusory depth from brightness arises from the complementary properties of boundary and surface processes, notably boundary completion and surface-filling in, within the parvocellular form processing cortical stream. This illusion depends upon how surface contour signals from the V2 thin stripes to the V2 interstripes ensure complementary consistency of a unified boundary/surface percept, while also enabling figure-ground separation to occur. Apparent motion of illusory contours arises from the complementary computations of form and motion processing across the parvocellular and magnocellular cortical processing streams. This illusion depends upon how illusory contours help to complete boundary representations for object recognition, how apparent motion signals can help to form continuous trajectories for target tracking and prediction, and how formotion interactions from V2-to-MT enable completed object representations to be continuously tracked even when they move behind intermittently occluding objects through time. Lages and Heron (2010) provide a mathematical analysis, from a purely formal perspective, of why fusion of depth and motion cues are “processed in parallel and integrated late in the visual processing hierarchy,” which they speculate to occur in MT or beyond, in order to solve the inverse problem of 3D motion perception.

Many other visual illusions have helped to discover, and been explained by, adaptive mechanisms whereby our visual brains achieve their unparalleled achievements in perceiving, understanding, and acting upon a rapidly changing world.

### Conflict of interest statement

The author declares that the research was conducted in the absence of any commercial or financial relationships that could be construed as a potential conflict of interest.
